# Application of clockwise modularized laparoscopic lymphadenectomy in the suprapancreatic area, a propensity score matching study and comparison with open gastrectomy

**DOI:** 10.1007/s00464-020-08070-w

**Published:** 2020-10-08

**Authors:** Hua Yang, Wei-Han Zhang, Kai Liu, Yu-Qing Dan, Xin-Zu Chen, Kun Yang, Zhi-Xin Chen, Jia-Ping Chen, Zong-Guang Zhou, Jian-Kun Hu

**Affiliations:** 1 State Key Laboratory of Biotherapy, Department of Gastrointestinal Surgery and Laboratory of Gastric Cancer, Collaborative Innovation Center for Biotherapy, West China Hospital, Sichuan University, No. 37 Guo Xue Xiang Street, Chengdu, Sichuan Province China; 2 West China School of Medicine, Sichuan University, Chengdu, China; 3 State Key Laboratory of Biotherapy, Department of Gastrointestinal Surgery and Laboratory of Digestive Surgery, Collaborative Innovation Center for Biotherapy, West China Hospital, Sichuan University, Chengdu, China

**Keywords:** Gastric cancer, Laparoscopy, Lymphadenectomy, Pancreas

## Abstract

**Background:**

Suprapancreatic lymphadenectomy is the essence of D2 radical gastric cancer surgery. The present study aimed to describe clockwise modularized laparoscopic lymphadenectomy in the suprapancreatic area.

**Methods:**

The data from gastric cancer patients who underwent surgical treatment from September 2016 to December 2018 were collected. Patients were divided into clockwise modularized lymphadenectomy (CML) and traditional open gastrectomy (OG) groups according to the surgical treatment strategy. The propensity score matching method was utilized to balance the baseline characteristics between the two groups.

**Results:**

Finally, 551 gastric cancer patients were included in the present study. Following propensity score matching, 106 pairs of patients in the CML group and OG group were included in the final analysis. The CML group had more total examined lymph nodes (36, IQR 28–44.74 vs. 29, IQR 29–39.5, *p* = 0.002) and no. 9 station nodes (2, IQR 1–5 vs. 2, IQR 1–3, *p* = 0.007) than the OG group. There was less intraoperative blood loss (30, IQR 20–80 ml vs. 80, IQR 50–80 ml, *p* < 0.001) and a longer surgical duration (262.5 min, IQR 220–303.25 min vs. 232, IQR 220–255 min, *p* < 0.001) in the CML group than in the OG group. The incidence of postoperative complications (19.8% vs. 16.0%, *p* = 0.591) and postoperative hospital stay (8, IQR 7–9 days vs. 8, IQR 7–9 days, *p* = 0.452) were comparable between the CML and OG groups.

**Conclusion:**

Laparoscopic lymphadenectomy for gastric cancer surgery is technically demanding. Clockwise modularized laparoscopic lymphadenectomy in the suprapancreatic area can attain similar effects as traditional open surgery and without an increase in postoperative adverse events.

**Electronic supplementary material:**

The online version of this article (10.1007/s00464-020-08070-w) contains supplementary material, which is available to authorized users.

The use of laparoscopic gastrectomy for gastric cancer patients has received increasing attention in recent years [1]. The short-term surgical safety and long-term oncological safety of laparoscopic gastric cancer surgery have been shown to be equivalent to those of traditional open surgery [1–3]. For advanced stage cancers, the short-term results from both the KLASS-02-RCT and JLSSG0901 trials confirm the noninferiority of laparoscopic gastrectomy to open gastrectomy [4, 5]. A recently reported study from the Chinese Laparoscopic Gastrointestinal Surgery Study (CLASS) group, the CLASS-01 trial, indicated that laparoscopic distal gastrectomy had comparable postoperative short-term adverse events and 3-year disease-free survival outcomes to open distal gastrectomy for locally advanced-stage gastric cancers [6, 7]. Therefore, the surgical and oncology safety of laparoscopic surgery for advanced-stage gastric cancer patients have been preliminarily verified.

Oncological safety is essential in surgical treatments of advanced gastric cancers. Several variables, such as tumor stage, genetic characteristics, and treatment strategy, can influence the survival outcomes of gastric cancer patients [8–10]. Given the limited operating space and lack of stereovision and haptic feedback, complete dissection of the regional lymph nodes is technically demanding in laparoscopic surgery. Laparoscopic lymphadenectomy increases the requirements for cooperation between the operator and assistants over those of traditional open gastrectomy. To facilitate and achieve thorough lymph node dissection, we established clockwise modularized laparoscopic lymphadenectomy strategies in a previous study [11]. However, the technical requirements of laparoscopic gastric cancer lymphadenectomy are even higher in the suprapancreatic area due to the deep anatomical location and variability of the vasculature this area. Meanwhile, achieving thorough lymphadenectomy in the suprapancreatic area without increasing perioperative complications is an essential demand of laparoscopic surgery for locally advanced gastric cancers. Therefore, we asked whether clockwise modularized laparoscopic lymphadenectomy could achieve the same effect as open surgery in suprapancreatic lymphadenectomy.

In the present study, we will present the details about the procedural demands and technical skills of lymphadenectomy in the suprapancreatic area in the clockwise modularized laparoscopic lymphadenectomy model and compare the clinical effect of these procedures with open gastrectomy in the same period by propensity score matching.

## Methods and materials

### Patients

The clinicopathological parameters of gastric cancer patients were retrieved from the database of the Surgical Gastric Cancer Patient Registry (SGCPR) in West China Hospital with the registration number WCH-SGCPR-2019-03 [12]. The use of this database in clinical studies or translational medicine research was approved by the Biomedical Ethical Committee of the West China Hospital, Sichuan University, China (No. 2014-215). Informed consent was obtained from the patients or their guardians. Patient records were anonymized and deidentified before analysis.

**Fig. 1:**
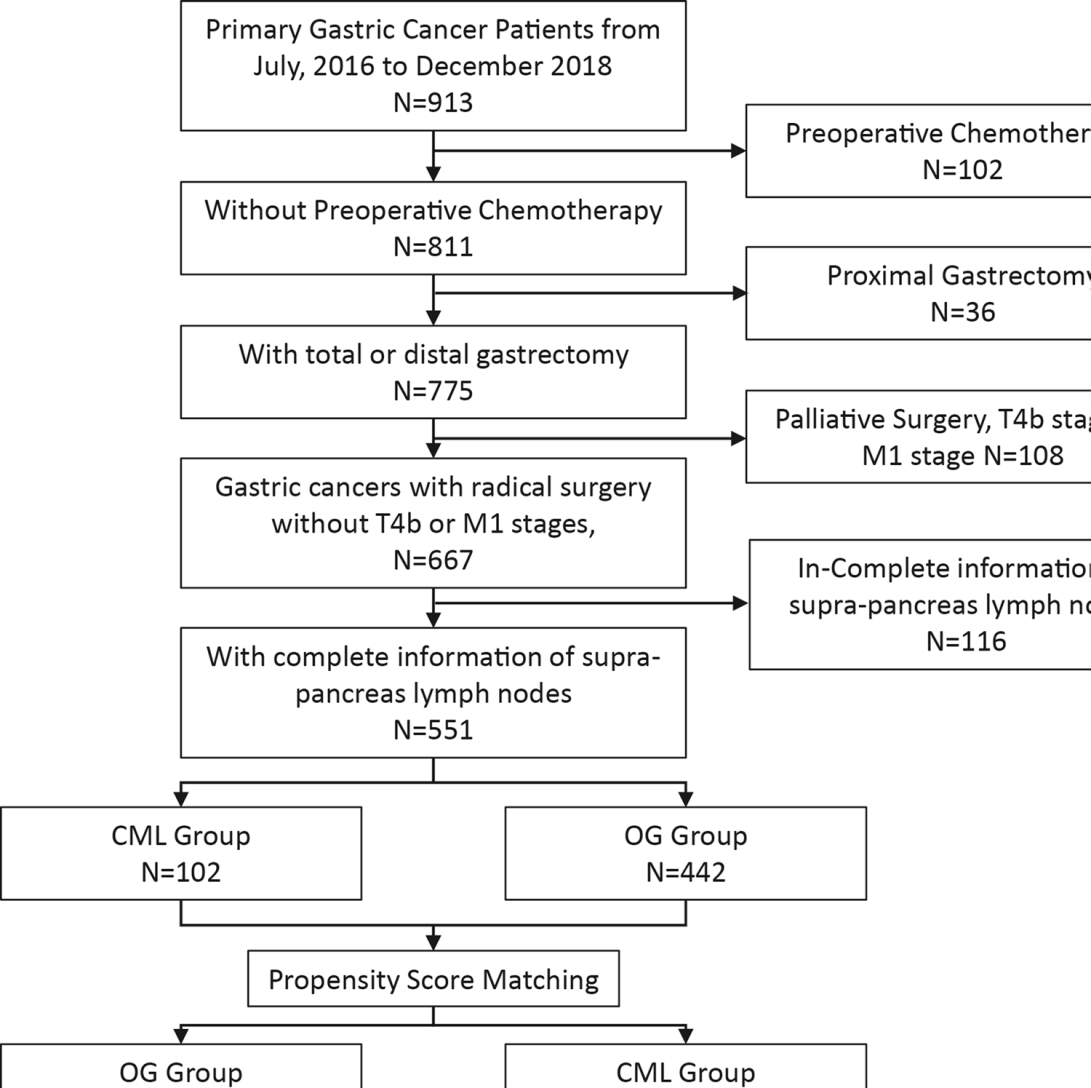
Flow chart of patient selection

From September 2016, our study group summarized the previous experience in laparoscopic surgery and established a standard method for performing clockwise modularized lymphadenectomy to facilitate laparoscopic surgery and promote thorough lymphadenectomy [11]. In this study, we collected gastric cancer patients from September 2016 to December 2018 who underwent radical gastrectomy from the SGCPR database. Patients who had preoperative chemotherapy, preoperative radiotherapy, or distant metastasis were excluded from the present study. Finally, according to the surgical treatment strategy, patients were assigned into a clockwise modularized laparoscopic (CML) group or an open gastrectomy (OG) group (Fig. 1).

### Clinicopathological characteristics

The following clinicopathological characteristics were assessed between the CML and OG groups. General characteristics, such as age (years), sex (male or female), body mass index (BMI) (kg/m^2^), tumor size (cm), tumor location, macroscopic type, resection types, and tumor stages, were used to estimate the baseline characteristics of the two groups. The operation-related outcomes, such as operation time (min), blood loss (ml), numbers of examined and metastatic lymph nodes (total, no. 7, no. 8a, no. 9, and no. 11p stations), postoperative hospital stay (days) and postoperative 30-day complications, were compared between the two groups.

Pathological examination was conducted by pathologists from the Department of Pathology, West China Hospital, Sichuan University according to the TNM Classification of Malignant Tumors, eighth edition, from the Union for International Cancer Control [13, 14]. Postoperative 30-day complications were defined as the complication incidence during the first 30 days of the postoperative period or complications occurring during the same hospitalization. The severity of postoperative complications was classified according to the Clavien-Dindo system [15].

### Surgical treatment

All patients underwent surgical treatment with radical intention in the Department of Gastrointestinal Surgery, West China Hospital, Sichuan University. The principles of the surgical treatment were based on the Japanese Gastric Cancer Treatment Guidelines [16]. Regardless of the use of either laparoscopic or open surgery, intraoperative frozen section examinations were routinely conducted to secure the safety of the resection margins. Early-stage gastric cancer patients were recommended to undergo laparoscopic surgery. The surgical indications of laparoscopic gastrectomy for locally advanced cancers were referred to the inclusion and exclusion criteria of the CLASS serious trials [6, 7, 17]. In particular, the use of laparoscopic gastrectomy or open gastrectomy for patients with advanced tumor stages were based on full communication between the surgeons and patients under the instructions of the Japanese Gastric Cancer Treatment Guidelines [16].

The detailed surgical procedures of clockwise modularized laparoscopic surgery were described in our previous study [11]. The core techniques of the procedure in the suprapancreatic area involve: (1) the nonpressed pancreas technique, (2) the overlook view technique, (3) the layer-by-layer reciprocal dissection procedure and (4) the application of pulling adventitia tissue skills. Details of the surgical procedures of clockwise modularized suprapancreatic lymphadenectomy are presented in Video 1.

**Fig. 2:**
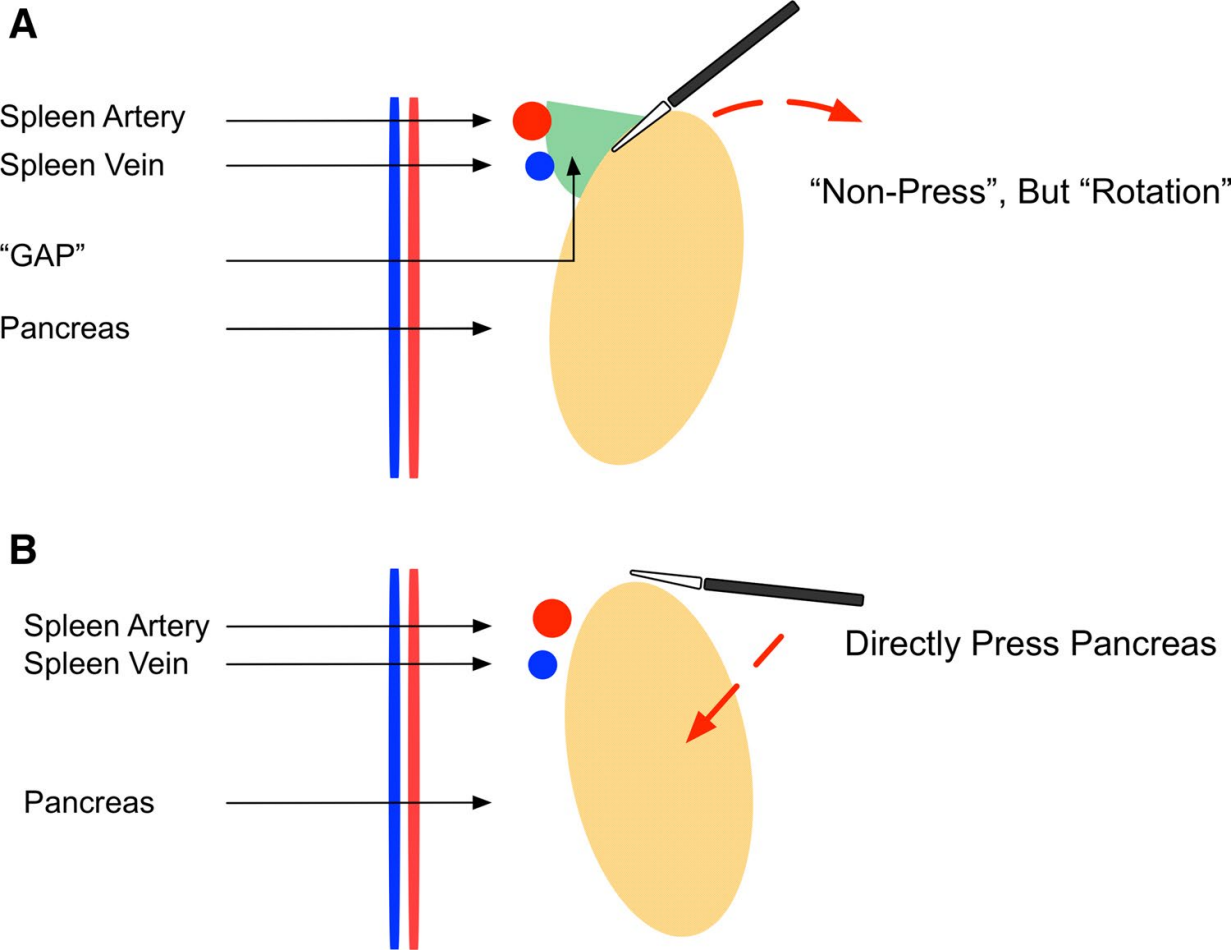
Illustration of the “nonpressed pancreas” technique (**A** Nonpressed pancreas, **B** Direct pressure on the pancreas)

**Fig. 3:**
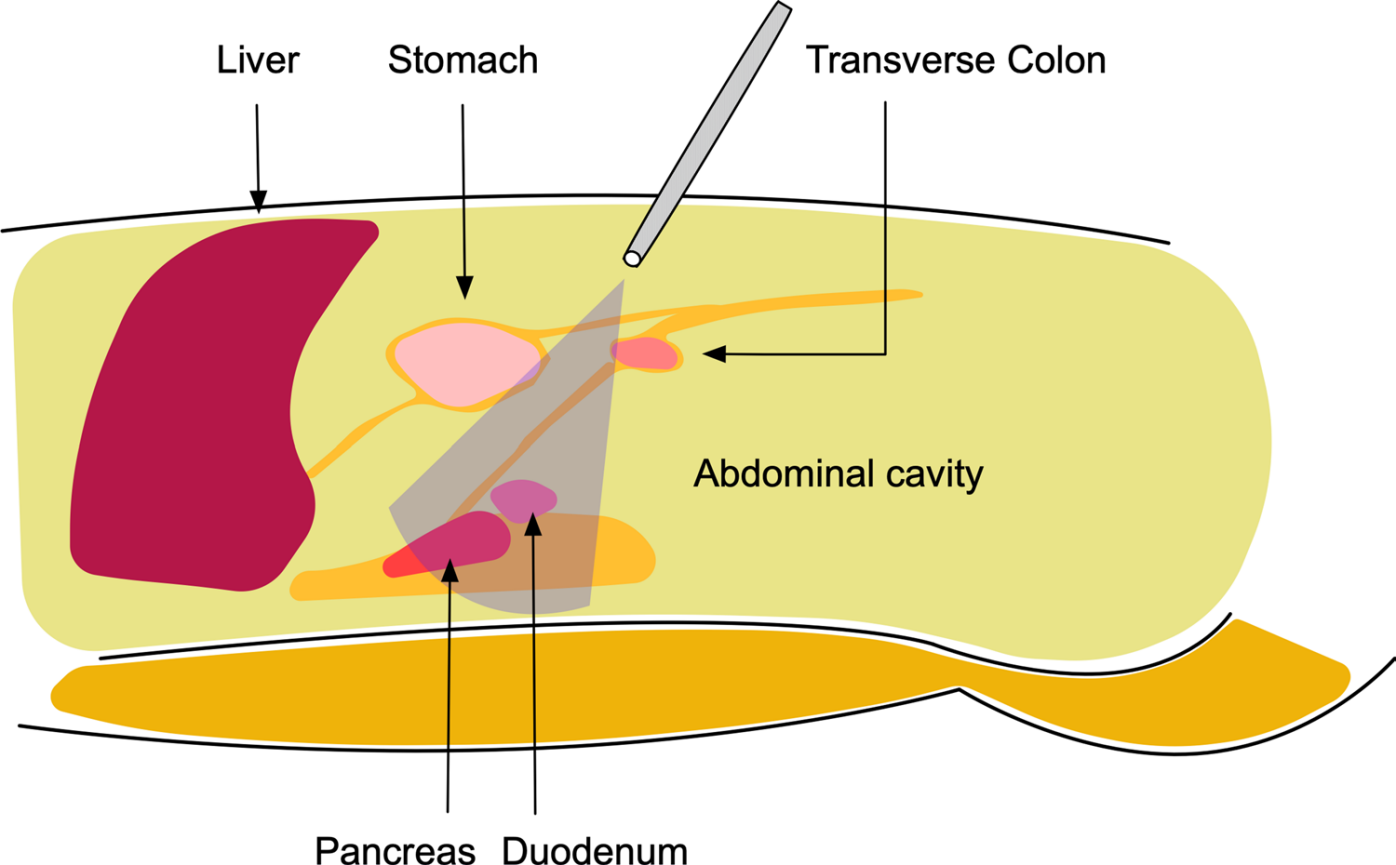
Illustration of the “overlook view” technique

**Fig. 4:**
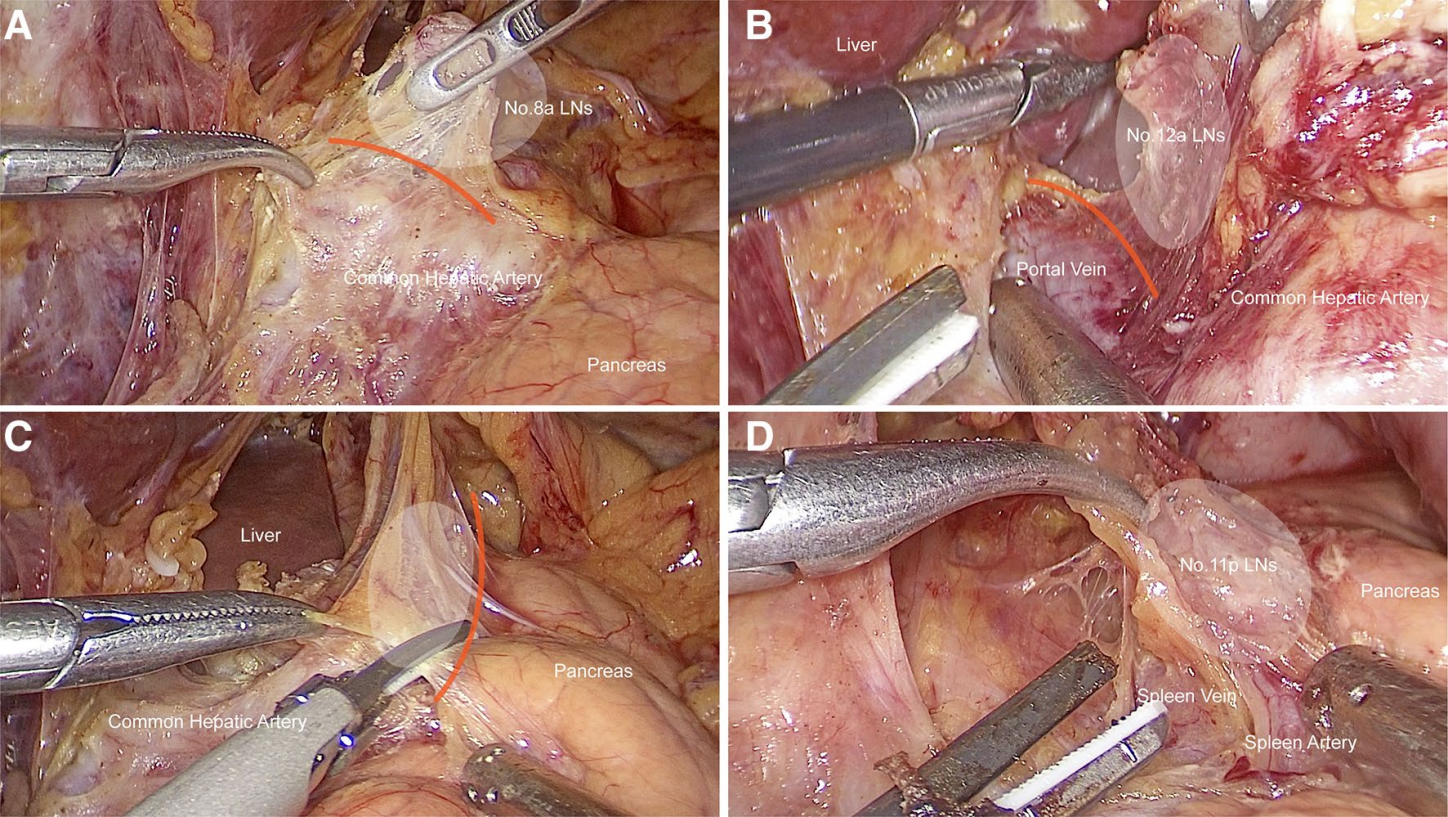
Intraoperative view of clockwise modularized laparoscopic suprapancreatic lymphadenectomy (**A** Right side of the suprapancreatic area (No. 8a LNs); **B** Right side of the suprapancreatic area (No. 12a LNs); **C** Left side of the suprapancreatic area (No. 7/11p LNs); **D** Left side of the suprapancreatic area (No. 11p LNs)

Specifically, we emphasize that the assistant should outwardly rotate the upper edge of the pancreas rather than directly deep press it (Fig. 2), and the laparoscope should look down on the suprapancreatic area by adjusting it to approximately 30° to acquire a similar view as traditional open surgery (Fig. 3). The application of these two skills was aimed at facilitating and securing the safety of suprapancreatic lymphadenectomy. The overlook view allows a clearer observation of the tissues in the back of the pancreas and reduces the possibility of accidental injury. The adoption of the nonpressed pancreas technique reduces direct damage to the pancreas from the laparoscopic instruments. The adoption of these skills can completely expose the spleen vein and portal vein, prevent injury to these vessels, and attain thorough lymph node dissection in the suprapancreatic area (Fig. 4).

### Statistical analysis

All statistical analyses were conducted with R Software Version 3.6.0 (https://www.R-project.org), including the “nonrandom” and “MatchIt” packages. Continuous variables with a normal distribution are presented as the means and standard deviations, and categorical variables are expressed as numbers (%). Medians and interquantile ranges (IQRs, p25-p75) were used to present continuous variables with abnormal distributions. The Mann–Whitney U test was utilized to analyze continuous variables and ordinal categorical variables, whereas the chi-square test was used for unordered categorical variables. A *P* value < 0.05 (2-sided) was defined as statistically significant. The propensity score (PS) was computed using a logistic regression model that included baseline characteristics (age, sex, tumor location, resection type, tumor size, T stages, and N stages) to balance the covariates between the CML and OG groups. Propensity score matching pairs were identified without replacement using a 1:1 nearest neighbor matching algorithm with caliper width determined by the recommendation (0.05 of the standard deviation of the logit) [18]. The balance of covariates between the groups was assessed by the standardized mean difference (SMD) before and after the matching procedures. An SMD < 0.1 indicated balance in the covariate between the two groups [19]. After the PS matching procedure, 106 matched pairs were generated with comparable characteristics.

## Results

### Clinicopathological characteristics

**Fig. 5:**
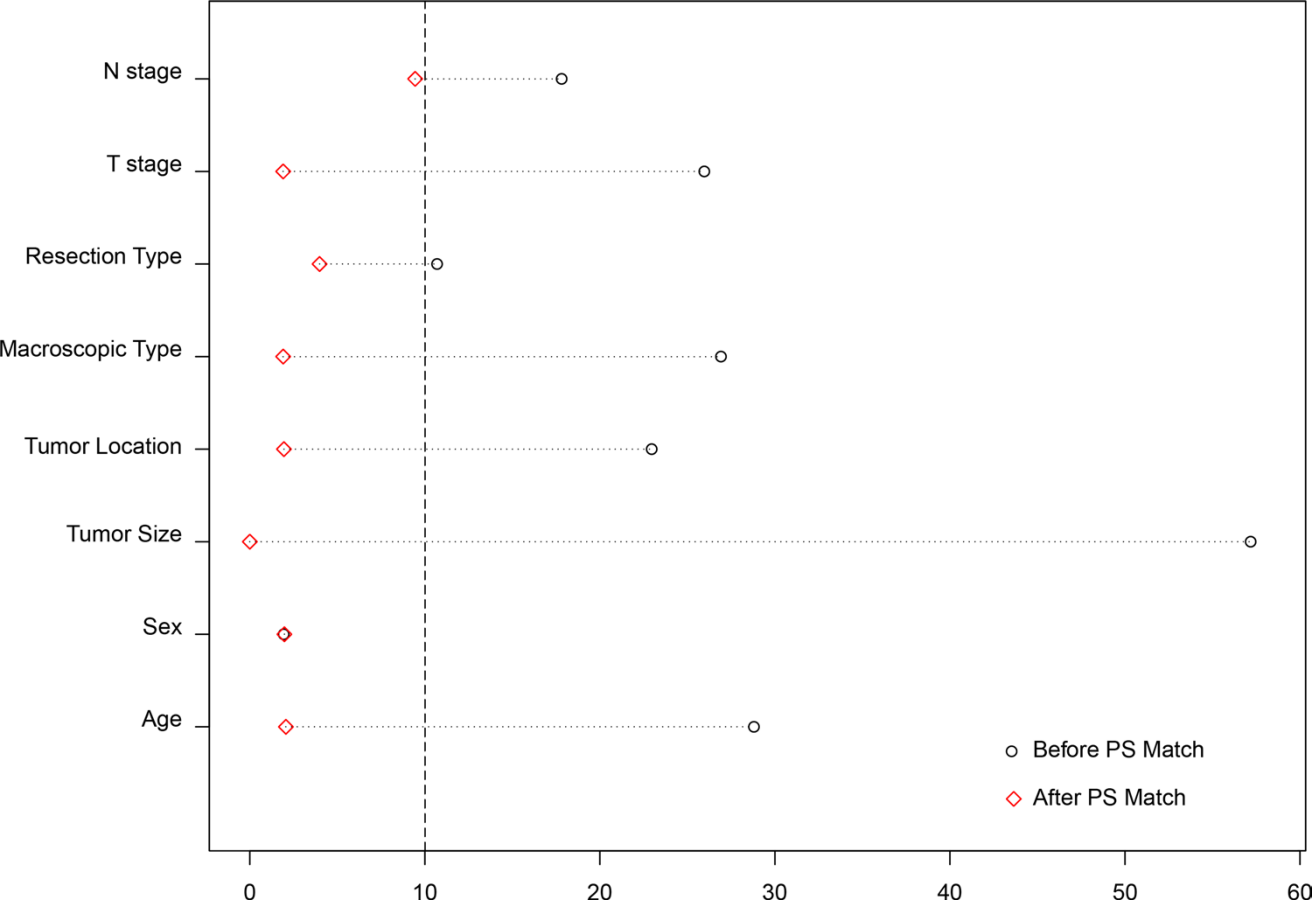
Standardized differences and distribution of baseline clinicopathological characteristics before and after propensity score matching. Age (Years); Sex (Male, Female); Tumor Size (< 4 cm and ≥ 4 cm); Tumor Location (adenocarcinoma of esophagogastric junction and Nonadenocarcinoma of esophagogastric junction; Macroscopic Type (Type 0–2 and Type 3–4); Resection Type (Distal gastrectomy and Total gastrectomy); T stage (T1 and T2-T4), and N stage (N0 and N1-3)

Clinical information from 551 gastric cancer patients who underwent radical surgical treatment was retrieved in the present study (Fig. 1). There were 109 patients in the CML group and 442 patients in the OG group. General clinicopathological characteristics are presented in Table 1. Before propensity score matching, five covariates (age, tumor size, tumor location, resection type, pT stage, and pN stage) were unbalanced (*p* < 0.05 or SMD > 0.1). After 1:1 PS matching, 106 matched pairs of patients were obtained with relatively balanced baseline characteristics between the two groups. The standardized differences and distributions of those characteristics before and after matching are presented in Fig. 5.

**Table 1:** The clinicopathological characteristics between the clockwise modularized laparoscopic group and open gastrectomy group in before and after propensity scoring matching cohort

Characteristics		Before Matching (*N* = 551)				After Matching (*N* = 212)			
		CML Group	OG Group	*P* value	SMD	CML Group	OG Group	*P* value	SMD
		*N* = 109 (%)	*N* = 442 (%)			*N* = 106 (%)	*N* = 106 (%)		
Age	Year	55.5 ± 10.7	58.7 ± 11.9	0.005	0.288	55.6 (10.7)	55.9 (13.0)	0.659	0.021
Sex	Male	72 (66.1)	296 (67)	0.946	0.019	69 (65.1)	68 (64.1)	1	0.020
	Female	37 (33.9)	146 (33)			37 (34.9)	38 (35.8)		
BMI level	Kg/m^2^	22.8 ± 3.1	22.9 ± 3.1	0.733	0.035	22.7 ± 3.1	22.4 ± 3.3	0.532	0.117
Tumor Size	< 4 cm	74 (67.9)	179 (40.5)	< 0.001	0.572	71 (67.0)	71 (67.0)	1	< 0.001
	≥ 4 cm	35 (32.1)	263 (59.5)			35 (33.0)	35 (33.0)		
Tumor Location	AEG	45 (41.3)	134 (30.3	0.039	0.230	42 (39.6)	43 (40.6)	1	0.019
	NonAEG	64 (58.7)	308 (69.7)			64 (60.4)	63 (59.4)		
Macroscopic Type	Type 0–2	63 (57.8)	312 (70.2)	0.014	0.269	60 (56.6)	61 (57.5)	1	0.019
	Type 3–4	46 (42.2)	130 (29.8)			46 (43.4)	45 (42.5)		
Resection Type	TG	38 (34.9)	132 (29.9)	0.37	0.107	35 (33.0)	37 (34.9)	0.885	0.04
	DG	71 (65.1)	310 (70.1)			71 (67.0)	69 (65.1)		
pT stage	T1	46 (42.2)	131 (29.8)	0.006	0.406	46 (43.4)	45 (42.5)	0.196	0.301
	T2	20 (18.3)	70 (15.9)			19 (17.9)	16 (15.1)		
	T3	31 (28.4)	127 (28.9)			29 (27.4)	22 (20.8)		
	T4	12 (11.0)	111 (25.3)			12 (11.3)	23 (21.7)		
	AGC	63 (57.8)	308 (70.2)	0.018	0.260	60 (56.6)	61 (57.5)	1	0.019
pN stage	N0	52 (47.7)	172 (38.9)	0.004	0.424	51 (48.1)	56 (52.8)	0.526	0.206
	N1	24 (22.0)	66 (14.9)			23 (21.7)	15 (14.2)		
	N2	20 (18.3)	81 (18.3)			19 (17.9)	19 (17.9)		
	N3	13 (11.9)	123 (27.8)			13 (12.3)	16 (15.1)		
	*N* ( +)	57 (52.3)	270 (61.1)	0.118	0.178	55 (51.9)	50 (47.2)	0.583	0.094

*CML* clockwise modularized laparoscopic lymphadenectomy; *OG* open gastrectomy; *SMD* standardized mean difference; *AEG* adenocarcinoma of esophagogastric junction; *TG* total gastrectomy; *DG* distal gastrectomy

### Clinical outcomes

The clinical outcomes were compared between the CML and OG groups, which included operation-related parameters (Table 2). In the after PS-matching cohort, although the CML group had a longer surgical duration than the OG group (262.5, IQR 220–303.25 min vs. 232, IQR 220–255 min, *p* < 0.001), it had less intraoperative blood loss than the OG group (30, IQR 20–80 ml vs. 80, IQR 50–80 ml, *p* < 0.001). The CML group had more total examined lymph nodes (36, IQR 28–44.74 vs. 29, IQR 29–39.5, *p* = 0.002), whereas there was no difference in the number of total metastatic lymph nodes (1, IQR 0–3 vs. 0, IQR 0–3.75, *p* = 0.965) between the two groups. In addition, there were more examined lymph nodes in the no. 9 station of the CML group than in the OG group (2, IQR 1–5 vs. 2, IQR 1–3, *p* = 0.007).

**Table 2:** The clinical outcomes between the clockwise modularized laparoscopic group and open gastrectomy group

Characteristics		Before Matching (*N* = 551)			After Matching (*N* = 212)		
		CML Group	OG Group	*P* value	CML Group	OG Group	*P* value
		*N* = 109	*N* = 442		*N* = 106	*N* = 106	
Operation Time	Min	260 (220–305)	235 (210–260)	< 0.001	262.5 (220–303.25)	232 (205–255)	< 0.001
Blood Loss	ml	30 (20–80)	80 (50–100)	< 0.001	30 (20–80)	80 (50–100)	< 0.001
Metastasis Lymph nodes (total)	Numbers	1 (0–3)	2 (0–7)	0.002	1 (0–3)	0 (0–3.75)	0.956
Examined Lymph nodes (total)	Numbers	36 (28–45)	29 (22–37.75)	< 0.001	36 (28–44.75)	29 (23–39.5)	0.002
Metastasis Lymph nodes (No.7)	Numbers	0 (0–0)	0 (0–0)	0.034	0 (0–0)	0 (0–0)	0.804
Metastasis Lymph nodes (No.8a)	Numbers	0 (0–0)	0 (0–0)	0.038	0 (0–0)	0 (0–0)	0.251
Metastasis Lymph nodes (No.9)	Numbers	0 (0–0)	0 (0–0)	< 0.001	0 (0–0)	0 (0–0)	0.110
Metastasis Lymph nodes (No.11p)	Numbers	0 (0–0)	0 (0–0)	0.048	0 (0–0)	0 (0–0)	0.556
Metastasis Lymph nodes (No.12a)	Numbers	0 (0–0)	0 (0–0)	0.011	0 (0–0)	0 (0–0)	0.243
Examined Lymph nodes (No.7)	Numbers	3 (1–4)	3 (2–5)	0.154	3 (1–4)	3 (1.25–4)	0.632
Examined Lymph nodes (No.8a)	Numbers	2 (1–3)	1 (1–2)	0.028	2 (1–3)	1 (1–2)	0.080
Examined Lymph nodes (No.9)	Numbers	3 (1.5–5)	2 (1–3)	< 0.001	2 (1–5)	2 (1–3)	0.007
Examined Lymph nodes (No.11p)	Numbers	1 (1–3)	1 (1–3)	0.529	1 (1–3)	2 (1–3)	0.215
Examined Lymph nodes (No.12a)	Numbers	1 (0–1.5)	1 (1–1)	0.516	1 (0–2)	1 (1–1)	0.844

*CML* clockwise modularized laparoscopic lymphadenectomy; *OG* open gastrectomy

### Postoperative outcomes

The postoperative-related outcomes compared between the two groups included postoperative hospital stays (days) and postoperative 30-day complications (Table 3). Interestingly, the CML group had a shorter length of postoperative hospital stay than the OG group in the before PS-matching cohort (8, IQR 7–9 days vs. 9, IQR 8–11 days, *p* < 0.001), and there was no significant difference between the two groups in the after PS-matching cohort (8, IQR 7–9 days vs. 8, IQR 7–9 days, *p* = 0.452). This may be due to the open gastrectomy group having more advanced tumor stage patients than the CML group in the before propensity score matching cohort. In addition, the CML group had comparable postoperative 30-day complications to the OG group in the after propensity score matching cohort (19.8% vs. 16.0%, *p* = 0.591), and the Clavien-Dindo classification showed no difference between the CML and OG groups (*p* = 0.697).

**Table 3:** The postoperative 30-day complications between the clockwise modularized laparoscopic group and open gastrectomy group

Characteristics		Before Matching (*N* = 551)			After Matching (*N* = 212)		
		CML Group	OG Group	*P* value	CML Group	OG Group	*P* value
		*N* = 109 (%)	*N* = 442 (%)		*N* = 106 (%)	*N* = 106 (%)	
Postoperative Stay	Days	8 (7–9)	9 (8–11)	< 0.001	8 (7–9)	8 (7–9)	0.452
Postoperative Complications	Yes	22 (20.2)	93 (21.0)	0.948	21 (19.8)	17 (16.0)	0.591
Clavien-Dindo Classification	Grade 1	18 (81.8)	81 (87.1)	0.474	18 (28.6)	16 (94.1)	0.697
	Grade 2	1 (4.5)	7 (7.5)		1 ( 1.6)	1 ( 5.9)	
	Grade 3	2 (9.2)	3 (3.2)		1 ( 1.6)	0 ( 0.0)	
	Grade 4	1 (4.5)	1 (1.1)		1 ( 1.6)	0 ( 0.0)	
	Grade 5	0	1 (1.1)		0	0	
Details of Complications	PPCs	20 (90.9)	76 (81.6)		19 (17.9)	16 (15.1)	
	Intraperitoneal Abscess	1 (4.5)	2 (2.2)		1 (0.9)	0	
	Anastomotic fistula	0	2 (2.2)		0	0	
	Gastroparesis	0	5 (5.4)		0	0	
	Ileus	1 (4.5)	3 (3.2)		1 (0.9)	1 (0.9)	
	Intraperitoneal hemorrhage	0	1 (1.1)		0	0	
	Upper gastrointestinal hemorrhage	0	2 (2.2)		0	0	
	Pancreatic fistula	0	1 (1.1)		0	0	

*CML* clockwise modularized laparoscopic lymphadenectomy; *OG* open gastrectomy *PPCs* postoperative pulmonary complications

## Discussions

Lymphadenectomy forms the basis of modern holistic treatment strategies for gastric cancer [20]. Laparoscopic gastrectomy presents a minimally invasive advantage over traditional open gastrectomy and is typically the focus of attempts to develop more advanced techniques. However, because of the limits of the abdominal space and the corresponding visual field, the technical demands for laparoscopic surgery have higher requirements for surgeons than those of open surgery. To improve the safety and efficacy of laparoscopic lymphadenectomy in gastric cancer surgery, we designed a clockwise modularized laparoscopic lymphadenectomy model based on the experience in our clinical practice [11]. This treatment strategy can examine more total lymph nodes with a shorter operation time and less intraoperative blood loss than traditional laparoscopic surgery [11]. Because of the deep anatomical location of suprapancreatic area lymph nodes, the distribution of important vessels and organs in this area, the limited abdominal space and visual field and the restricted manipulability of the tools, laparoscopic suprapancreatic lymphadenectomy is difficult, and the procedures have high technical demands. In clockwise modularized lymphadenectomy, we scheduled technical demands and skills for both the surgeons and assistants. In comparison with traditional open gastrectomy, we found that clockwise modularized lymphadenectomy can achieve a similar effect on lymph node resection in the suprapancreatic area without an increase in surgical adverse events.

Suprapancreatic lymph node dissection is one of the core elements of D2 gastrectomy [21, 22]. The deep anatomical locations of the lymph nodes and tendencies of the patients to have high BMI and advanced tumor stages raise the technical difficulty of dissecting these lymph nodes [23, 24]. In the present study, BMI (*p* = 0.532, SMD = 0.117) and tumor stage (pT stage, *p* = 0.196, SMD = 0.301; pN stage, *p* = 0.526, SMD = 0.206) were balanced by PS matching between the two study groups. Considering that the SMD of pT stage and pN stage was higher than 0.1 in the after PS match cohorts, we calculated the *p* value and SMD between the two groups for advanced gastric cancer (T2-T4 stages, *p* = 1, SMD = 0.019) and nodal positive patients (*p* = 0.583, SMD = 0.094). This was due to the limitations of the retrospective study, selection bias, and tumor stage differences in the selection of laparoscopic or open gastric cancer surgery. According to the recommendations from the Japanese Gastric Cancer Treatment Guidelines [16], patients who are screened for laparoscopic surgery in our center are informed of the details about the clinical tumor stage, current guidelines and clinical evidence about laparoscopic gastric cancer surgery. Then, treatment selection is decided by the patient after full communication with the surgeons. Therefore, although propensity score matching was used in the statistical analysis, because advanced stage patients prefer open surgery, the tumor stage still cannot perfectly match in the after PS match cohort.

The safety of laparoscopic gastrectomy has been proven by several previous studies for both laparoscopic distal and total gastrectomy [6, 7, 25]. We noticed that the incidence of postoperative 30-day complications was comparable between the CML and OG groups, and postoperative pulmonary complications were more common in the CML group than in the OG group (19/106 vs. 16/106). A longer surgical duration accompanied by longer anesthesia time and longer-term tracheal intubation may increase the risk of postoperative pulmonary complications [26]. Meanwhile, the severity of postoperative complications according to the Clavien-Dindo classification was also comparable between the two groups both before and after propensity score matching. Therefore, clockwise modularized supra-pancreas lymph node dissection does not increase the risk of postoperative complications compared with traditional open surgery. We also noticed that in the KLASS-01 and KLASS-02 studies, laparoscopic surgery had a lower incidence of postoperative complications and a shorter postoperative hospital stay [27, 28]. However, the CLASS-01 study indicated that there is no difference in postoperative complications between laparoscopic surgery and open surgery [7]. Several factors, such as tumor stage, resection pattern, anesthesia strategy and perioperative management strategy, can influence the incidence of complications and length of postoperative hospital stay. Therefore, laparoscopic surgery is at least equal to or better than open surgery in terms of short-term outcomes according to the present clinical evidence.

The number of examined lymph nodes is an important indicator for evaluating the quality of gastric cancer surgery. Currently, more than 25 examined lymph nodes is recommended for advanced gastric cancers or nodal positive cancers [29, 30]. Higher numbers of examined lymph nodes indicate the low potential of a false-negative lymph node rate and may result in better survival outcomes [31]. Our previous study successfully demonstrated that clockwise modularized lymphadenectomy has a clinical advantage in lymph node dissection over traditional laparoscopic surgery [11]. In the present study, we found that the CML group had more total examined lymph nodes than the OG group. In the suprapancreatic area, the number of no. 9 station nodes was higher in the CML group than in the OG group. These results showed that clockwise modularized lymphadenectomy has an advantage in suprapancreatic lymph node dissection over traditional open gastrectomy.

From the aspect of operational technology and skill, the clockwise modularized method facilitates laparoscopic lymphadenectomy in the suprapancreatic area. First, the assistants make full use of the 30° angle-adjusting function of the laparoscope, obtaining a visual field for overlooking the target (the suprapancreatic area) similar to that in traditional open surgery. This can reduce the risk of accidental injury to adjacent organs or tissues. In addition, we emphasize that the energy device must be under the view of the laparoscope and ascertain the safety of adjacent tissues. Pancreas fistula is a postoperative complication of gastric cancer surgery that can be caused not only by accidental injury from the energy device but also by direct traumatic injury from the laparoscopic forceps [32]. In the present study, the incidence of pancreatic fistula was low in both the CML group and the OG group. This is due to the advantage resulting from the adoption of the overlook view skill and the nonpressed pancreas technique during the laparoscopic operation. The overlook view skill provides a better visual field of view, and the nonpressed pancreas technique can be performed by the assistant to avoid direct injury to the pancreas from the laparoscopic instruments. In the nonpressed pancreas technique, the assistant gently pushes on the upper edge of the pancreas with the forceps instead of directly pressing deeply onto the pancreas. This technique can result in eversion of the pancreas, bringing the suprapancreatic lymph nodes closer to the laparoscope. The extorsion effect can increase the dissection space and generate better tissue tension in the gap between the splenic artery and the edge of the pancreas, which facilitates the dissection of lymph nodes along with the spleen and artery and reduces the potential of injury to these vessels. Last, the lymphadenectomy in the suprapancreatic area should be performed sequentially from individual points to surfaces and finally to the entire three-dimensional space, rather than alone in-depth in one station. For example, we first establish the left gastric vessels as the central landmark, then clear the membrane of the plica gastropancreatica from the right to left side, and finally completely removed lymphatic tissue in these areas. Subsequently, we can obtain a clearer anatomical space and view and avoid accidental injury during the operation. Therefore, the benefits of clockwise modularized suprapancreatic lymph node dissection are a similar radical degree of lymph node dissection of the suprapancreatic area, similar surgical safety, and a similar risk of postoperative complications as open surgery.

The emergence of 3-dimensional and 4 K laparoscope instruments can offer us stereoscopic vision and even better views of the surgical areas. Fortunately, the surgeons can continue to expand their repertoire of surgical skills, guaranteeing operational safety and benefit to the patients. Clockwise modularized laparoscopic lymphadenectomy is the result of our experience in clinical practice on gastric cancer surgery. We believe that with the continued accumulation of surgical experience, better surgical techniques and strategies will be proposed based on our present clockwise modularized model.

## Conclusions

Laparoscopic lymphadenectomy for gastric cancer surgery is technically demanding. Clockwise modularized laparoscopic lymphadenectomy in the suprapancreatic area can attain similar effects without increases in postoperative adverse events as traditional open surgery.

## Electronic supplementary material

Below is the link to the electronic supplementary material.

Video 1. Application of clockwise modularized laparoscopic lymphadenectomy in the suprapancreatic area. Supplementary file1 (MP4 238996 kb)

## Abbreviations

CML: Clockwise modularized lymphadenectomy
OG: Open gastrectomy
RCTs: Randomized control trials
CLASS: Chinese Laparoscopic Gastrointestinal Surgery Study
SGCPR: Surgical Gastric Cancer Patient Registry
BMI: Body mass index
